# Super-Resolution Reconstruction of Remote Sensing Images Using Multifractal Analysis

**DOI:** 10.3390/s91108669

**Published:** 2009-10-29

**Authors:** Mao-Gui Hu, Jin-Feng Wang, Yong Ge

**Affiliations:** Institute of Geographic Sciences & Nature Resources Research, Chinese Academy of Sciences, Beijing, China; E-Mails: humg@lreis.ac.cn (M.H.); gey@lreis.ac.cn (Y.G.)

**Keywords:** super-resolution reconstruction, multifractal analysis, information transfer, fractal code, gaussian upscaling

## Abstract

Satellite remote sensing (RS) is an important contributor to Earth observation, providing various kinds of imagery every day, but low spatial resolution remains a critical bottleneck in a lot of applications, restricting higher spatial resolution analysis (e.g., intra-urban). In this study, a multifractal-based super-resolution reconstruction method is proposed to alleviate this problem. The multifractal characteristic is common in Nature. The self-similarity or self-affinity presented in the image is useful to estimate details at larger and smaller scales than the original. We first look for the presence of multifractal characteristics in the images. Then we estimate parameters of the information transfer function and noise of the low resolution image. Finally, a noise-free, spatial resolution-enhanced image is generated by a fractal coding-based denoising and downscaling method. The empirical case shows that the reconstructed super-resolution image performs well in detail enhancement. This method is not only useful for remote sensing in investigating Earth, but also for other images with multifractal characteristics.

## 1. Introduction

Super-resolution (SR) reconstruction is an attractive and promising method in digital image processing that aims at producing a detailed and spatial resolution-enhanced image from one or more low-resolution (LR) images [1,2]. Depending on the number of LR images involved, the SR method can involve multi-frame or single-frame SR reconstruction [3]. The former tries to combine complementary information from different images based on sub-pixel shifts or different parameter information, while the latter focuses on extracting relationships among neighborhood pixels or learning *a priori* pattern structures from image databases that store large amounts of low-high resolution image pairs [4-8]. In practice, multi-frame SR is usually applied in video and multi-sensor observation, since multiple frames can be easily obtained. It is difficult, however, for some satellite remote sensing to get several images of the same scene in a short time, especially for highly dynamic scenes. Therefore, sub-pixel unmixing-based, single-frame SR image mapping has become a popular topic in remote sensing and has been applied in many kinds of SR mapping [9-13]. It first estimates fractions of each endmember (pure component in the image) in pixels and then finds out the position of each endmember with spatial correlations or *a priori* knowledge. In the method, the number of endmembers should be greater than one. In atmosphere observation, trace gas distributions, for example, often change greatly within hours and are not as stable as landscape observations like land cover types whose states remain unchanged for several days or even months. Different observation times have different views, thus the traditional multi-frame SR is not suitable for such changeable images. The sub-pixel unmixing SR mapping method is invalid for images whose pixel value cannot be separated from endmembers, such as elevation, surface temperature, and trace gases density.

Fractal theory is a very efficient method to depict chaotic, erratic, natural phenomena. After its conception in the 1970s by Mandelbrot, fractal theory was applied to numerous domains [14,15]. It is considered an appropriate and straightforward method to analyze not only the scale independency of geophysical, observable things, but also the extreme variability over a wide range of scales [16]. It permits the characterization of complex phenomena in a fully quantitative fashion. The fractal coding method was originally applied by Barnsley *et al.* to image compression based on the Iterated Function System theory (IFS) [14]. Since it is difficult to implement the original IFS method in practice, Jacquin proposed an automatic grayscale still image coding method, Partitioned IFS (PIFS), that partitioned the whole image into smaller segments [17]. The most important precondition of fractal image coding is that the object images have the characteristic of self-similarity or self-affinity. It is widely accepted that many natural scenes and images have fractal/multifractal characteristic, e.g., trees, clouds, and mountains [18]. Therefore a great deal of redundant information exists in these images, which can be interpreted by a contractive fractal transform operator *W* that consists of a geometrical transformation and a gray-level (also luminance) transformation on images. Then, only parameters of the transformation need to be stored. The original images can be reconstructed by the attractive fixed point of the operator *W* guaranteed by the Collage theorem. Fractal image coding has aroused great research interest since its birth in the 1990s, and most is concentrated on the compression ratio and speed improvement of image compression [19,20]. In this paper, we propose a super-resolution reconstruction and mapping method based on multifractal analysis and coding theory. It does not need *a priori* information or data.

The remainder of the paper is organized as follows. In Section 2, we present detailed methods for reconstructing super-resolution images based on multifractal analysis and coding. We first investigate multifractal methods in order to better explore the multifractal characteristic of images. Then we propose the fractal-based super-resolution reconstruction method that consists of parameter estimation, denoising, and downscaling. An empirical study is presented in Section 3. Finally, we discuss the results and draw conclusions in Section 4.

## 2. Methods

**Figure 1.**
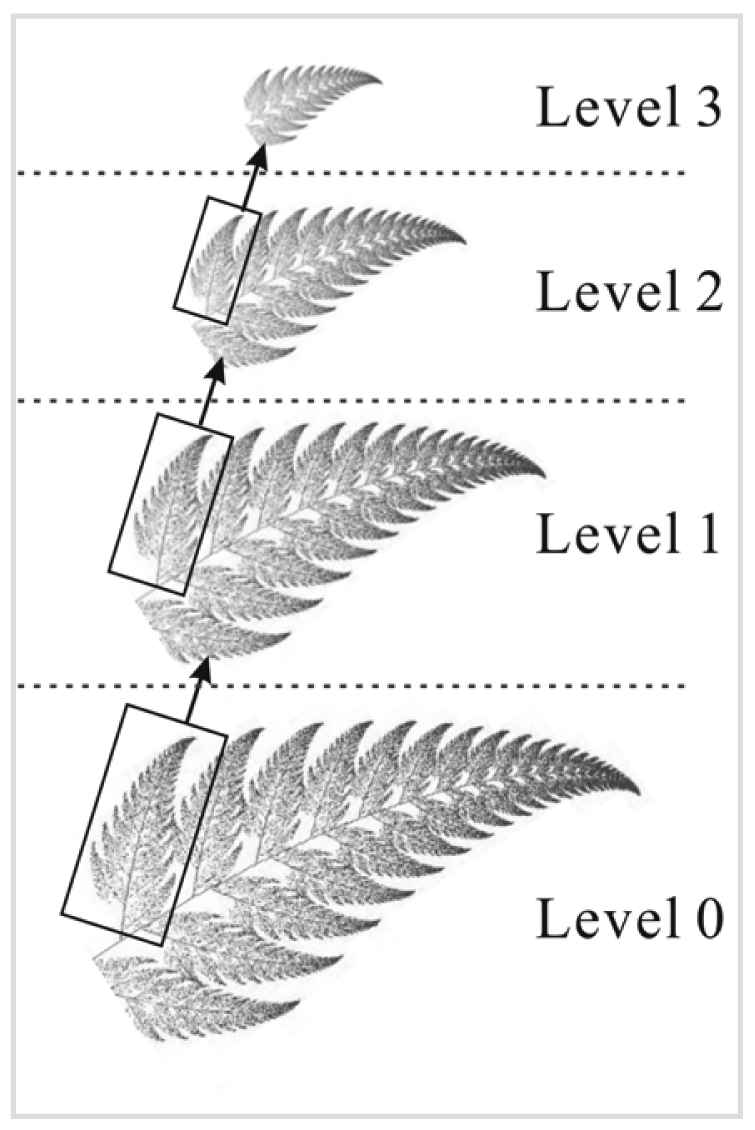
Self-similarity between scales.

The relationship between a high spatial resolution image *H* and the corresponding low spatial resolution image *L* could be presented by a multiplication process between *H* and information transfer function *s*, which expresses how information is transferred between different scales [21]:

(1) L=H⊗s+e

where ⊗ denotes the multiplication operator and *e* is the noise. In this paper, we take *e* as additive white Gaussian noise (AWGN), one of the most common cases in practice. The function *s* moves through *H* continuously with no overlap. Then, the super-resolution reconstruction problem becomes how to get *H* under the condition of *L, s*, and *e*. For an arbitrary *L*, there may be numerous *H* that can generate *L* with the same *s*. However, given *L, s*, and *e, H* could be determined uniquely if the original image/scene has fractal/multifractal characteristic, meaning self-similarity or self-affinity characteristics are present in the parts and the whole at different scales (Figure 1). With multifractal analysis, we explore the upscaling information transfer function *s* in a natural scene image from large scale to small scale, and estimate the additive white Gaussian noise distribution *e* in the image. Then, we reconstruct a SR image from a single LR image by denoising and downscaling.

**Figure 2.**
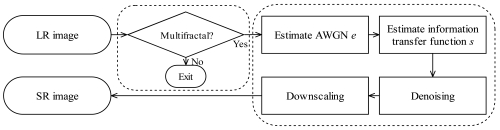
Framework of SR construction.

The framework of super-resolution reconstruction using multifractal analysis is shown in Figure 2. In the method, a multifractal characteristic is required to reconstruct an SR image, so this aspect was explored first. Then we estimated parameters of the additive white Gaussian noise distribution *e* and the information transfer function *s*. Lastly, an SR image was reconstructed by denoising and downscaling in a fractal coding process.

### 2.1. Multifractal Analysis

Fractal dimension is a basic tool of fractal theory to quantify irregular patterns or behaviors in natural physical systems. It reflects the extent of a measure's smoothness or roughness quite well. For fractal objects, the relationship between a certain size and the number of objects can be expressed as [22]:

(2) $$N(ɛ)∼{ɛ}^{-D}$$

where *ε* is the scale, *N*(*ε*) is the number of objects, and *D* is the fractal dimension. The equation shows the power law relation between the scale and the number of objects. Fractal dimension characterizes the average properties of a system and cannot provide information on deviations from the average behavior of a power law. When calculating the size of *N*(*ε*) in the box counting method, a box is considered to be either empty or occupied, ignoring the mass density variation in boxes. Thus, it is not enough to characterize a system with non-homogeneous or non-isotropic scaling properties. More scaling exponents and fractal dimensions are needed to assess it. For such a complex system one could resort to multifractal analysis, which adopts a continuous spectrum of exponents for the characterization of a system. Covering the support of the measure with boxes of size *l* and accounting for the mass probability (*p_i_*) in the *i*-th box, an exponent *α_i_* (singularity strength) can be defined by [23]:

(3) $$p_{i}(l)∼l^{\alpha_{i}}$$

Given *N*(*α_i_*) is the number of boxes with the same probability *p_i_*, we can define *f*(*α*) (multifractal spectrum) as the fractal dimension of the subset of boxes with exponent *α* by: *N_α_*(*l*)∼*l*^–^*^f^*^(^*^α^*^)^, which generalizes Equation (2) by including several indices to quantify the scaling of the system.

A multifractal complex system can be decomposed into a series of subsets with different *α*, and *f*(*α*) is such a cluster that represents the subsets' fractal dimensions. For a multifractal measure, plotting *α-f*(*α*) yields a concave downward function with a unimodal appearance.

Besides the singularity spectrum, the generalized dimension *D_q_* is another important index to describe the singular measure through scaling the *p_i_* distribution moments in the form [23]:

(4) $$\sum_{i=1}^{N(l)}p_{i}^{q}=l^{\tau (q)},\tau (q)=(q-1)D_{q}$$

where *p_i_* is the probability of the measure in the *i*-th box; *q* is the moment order (-∞ < q < ∞), *D_q_* is the generalized fractal dimensions, and *τ*(*q*) is the correlation exponent of the *q*-th order moment. For a multifractal measure, the generalized dimension *D_q_* is strict monotonous decreasing functions. Specially, when *q* takes a value of 0, 1, or 2, we can get capacity dimension *D*_0_, entropy dimension *D*_1_, and correlation dimension *D*_2_, respectively. The relationship between them is *D*_0_ ≥ *D*_1_ ≥ *D*_2_, where equality occurs when the measure is mono-fractal [24].

### 2.2. Super-Resolution Reconstruction

Besides its high compression ability, fractal coding has some important properties for image resolution enhancement [25]: (1) Resolution independence: after being converted to fractal code, the image code is resolution independent and, theoretically, digital images of any resolution can be generated at the decoding step. The infinite scaling property is also called “fractal scaling”. (2) Similarity preservation: in fractal-generated images, some similarities can be preserved at different scales. (3) Nonlinear operation: fractal coding is an adaptive, locally linear, yet globally nonlinear method and is beneficial for restoring missing details for resolution enhancement. Because of fractal coding's flexibility, some researchers process images for purposes beyond compression, such as image interpolation, image zooming, and image restoration and denoising. Ghazel *et al.* proposed a fractal-based method to restore noise-free images from noisy images by establishing a relationship between the fractal code of the original noise-free image and the noisy counterpart based upon some knowledge of the noise [26]. Chen *et al.* proposed super-resolution image reconstruction in Discrete Cosine Transform (DCT) domain [25]. To the best of our knowledge, however, in most fractal coding research and applications the shrinking operation of geometrical transformation is achieved by either downsampling by taking every *n* pixels or averaging over *n* by *n* pixels. This strategy is acceptable in common fractal coding applications, such as image compression and texture segmentation. But, when it comes to resolution enhancement or super-resolution reconstruction, it is imprudent to ignore the fact that physical systems that exhibit chaotic or fractal behavior in nature lose information exponentially between different scales [23,27]. It is important to consider a proper information transfer function in the fractal image coding process. In this study we concentrated on the general upscaling process, and explored the relationship between a noise-free image and a noisy image under the general framework. Then a fractal coding-based image restoration and super-resolution reconstruction method was proposed. The main flow of the SR image reconstruction contains the following steps: (a) parameter estimation, including the information transfer function and noise distribution; (b) fractal image encoding and image restoration (noise-free PIFS codes and the image were generated from the corresponding noisy image); (c) upscaling the LR image with a magnification coefficient.

#### 2.2.1. Information transfer function (ITF)

**Figure 3.**
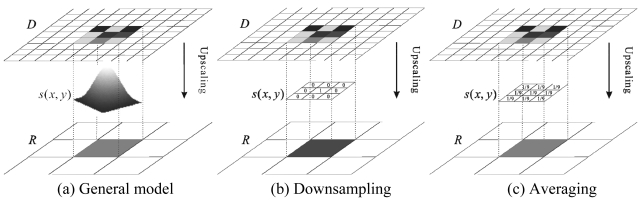
Information transfer function *s*.

The information transfer function *s* measures the information transfer mechanism between different scales in nature (Figure 3). It describes how information is preserved and lost in the upscaling process, where large textures and shapes are preserved while small details are eliminated because of synthesis. From the view of optical imaging, the information transfer function (ITF) has a close relationship with the point spread function (PSF), *s*(*I*) = *S*_↓_(*f*(*I*)), where *f*(·) is the point spread function and *S*_↓_(·) is the downsampling function.

It is worthwhile to explore the relationship between the information transfer function and traditional downsampling and averaging shrinking methods. Given *ω* is a discrete template of the information transfer function *s*(·): (1) If *ω_k_* = 1, *ω_j_* = 1, *j* ≠ *k*, where (*j, k* = 1, 2, …, *n*^2^), this is the down-sampling shrinking method (Figure 3b); (2) If *ω_j_* = 1/*n*, where (*j* = 1, 2, …, *n*^2^), then the shrinking method is averaging (Figure 3c). Downsampling and averaging shrinking methods are two special cases of the information transfer function. Thus, the relationship between the ITF and traditional downsampling and averaging shrinking methods is generalization and specialization.

In practice, getting the information transfer function *s* is quite challenging, especially when there is little *a priori* knowledge of how information is exhibited and changes in different spatial scales. It is widely accepted that physical systems lose information at an exponential rate [23]. Furthermore, the Gaussian pyramid has played an important role in a wide range of image visualizations and is consistent with the visual characteristics of human perception [28-30]. A Gaussian function would be an optimal approximation of the process of information transfer in upscaling. A typical expression of a 2D Gaussian function is formed as:

(5) $$G(x,y)=\frac{1}{2\pi \delta^{2}}e^{-\frac{x^{2}+y^{2}}{2\delta^{2}}}$$

where *x* is the distance from the origin in the horizontal axis, *y* is the distance from origin in the vertical axis, and *δ* is the standard deviation of the Gaussian distribution (Figure 3a). Gaussian upscaling can also be viewed as a generalized form of averaging when *δ* → 0 and downsampling when *δ* → ∞. Especially in discrete space, a Gaussian upscaling template is the same as an averaging template when the size of the domain block is four times the size of the range block in fractal coding, where domain block and range block are some suitable partition for the object image (see Section 2.2.2 for details).

#### 2.2.2. Fractal encoding

Let *I* denote an image of interest defined by an image function *u*(*x, y, p*), where (*x, y*) is the pixel coordinate of the image and *p* is the intensity value of the pixel. *R* and *D* are range blocks and domain blocks of image *I*, respectively, which are a kind of suitable partition for the image. Each range block *R_i_*∈*R* (*i* = 1, 2, …, *M, M* is the number of range blocks) is associated with a domain block *D_j_*∈*D* (*j* = 1, 2, …, *N, N* is the number of domain blocks) by a contractive mapping *w_j_*, which consists of two transformations: a geometrical transformation *g_ij_*: *D_j_* →*R_i_* (*R_i_, D_j_* ⊂ Ω, Ω denotes the space of image gray level; *i* = 1, 2, …, *M; j* = 1, 2, …, *N*) and a gray-level transformation *ϕ_j_* : *Θ* →*Θ* (*Θ* is the set of real number) [31]:

(6) $$\forall (x,y,p)\in D_{j},w_{j}(x,y,p)=(g_{ij}(x,y),\varphi_{j}(p))$$

where *i* = 1, 2, …, *M; j* = 1, 2, …, *N*.

Generally, the geometrical transformation *g_ij_* can be formed as:

(7) $$g_{ij}(\cdot )=s_{ij}(r_{ij}(\cdot ))$$

where *r_ij_*(·) is an affine mapping operator to make a domain block *D_j_* similar to a range block *R_i_* and *s_ij_*(·) is a shrinking operator to make the domain block *D_j_* have the same size with the range block *R_i_*. In a uniform partitioning scheme, *R_i_* and *D_j_* are usually square pixel blocks, and the size of *D_j_* is several times the size of *R_i_*. In discrete cases, eight affine mapping operators are often used, namely, a horizontal flipping, a vertical flipping, two diagonal flippings, and four rotations (±90°, and ±180°). The luminance transformation, also called gray-level mapping, is a first-order linear prediction of the form [26]:

(8) φ(t)=αt+β

where *ϕ*(*t*) and *t* are the intensities of the pixels within *R_i_* and g*_ij_*^(^*^k^*^)^(*D_j_*) (*k* denotes the *k*-th affine mapping operator), respectively; *α* is a scalar factor; and *β* is a transform term. The parameters *α* and *β* can be determined by minimizing the following collage error according to the Collage theorem [25]:

(9) $$\Delta_{ij}^{\left(k\right)}={\left\|\alpha_{ij}g_{ij}^{\left(k\right)}(D_{j})+\beta_{ij}-R_{i}\right\|}_{2}$$

where *k* denotes the *k*-th affine mapping operator; the norm ‖·‖_2_ calculates the Euclidian distance between the transformed domain block g*_ij_*^(^*^k^*^)^(*D_j_*) and the range block *R_i_*.

Thus, the fractal code of *R_i_* can be represented by a five-element set (*i, j, k, α_ij_, β_ij_*), where *i, j*, and *k* are the indexes of range block *R_i_*, domain block *D_j_*, and affine mapping operator *r_k_*(·), respectively; and *α_ij_* and *β_ij_* are the corresponding coefficients of collage error. All these sets of range blocks together are called the PIFS code of image *I*. In fractal coding, the overlapped range block partition method is sometimes adopted to avoid blocky artifacts and to capture finer details. This increases computation time, however, and requires more memory.

#### 2.2.3. Image denoising

The process of upscaling from domain block *D_j_* to range block *R_i_* is a multiplication operation between information transfer function *s* and *D_j_*. The function *s* denotes information transfers from the domain blocks scale to the range blocks scale. The formalism presents the shrinking process: *r*(*x, y*) = *s*(*d*(*x, y*)), where *r* and *d* are two images of the same scene at the range block scale and domain block scale, respectively (Figure 3). Then, the density value of pixel *v* within *R_i_* can be expressed by a linear expression:

(10) $$v=\sum_{i=1}^{n\times n}\omega_{i}\lambda_{i}$$

where *λ_i_* (*i* = 1, 2, …, *n*^2^) are density values of pixels within *D_j_* that are contained within the extent of *R_i_*, and *ω_i_* (*i* = 1, 2, …, *n*^2^) are the discrete values of the shrinking function in discrete space, subjected to Σ*ω_i_* = 1 (0 ≤|*ω_i_*|≤ 1).

Ghazel *et al.* restored noise-free images from noisy images based on fractal coding with an averaging operation method to produce the transformed block from the parent block of the same size as the child block [26]. As mentioned before, the upscaling information transfer function usually has a more complicated form rather than averaging operations. With the analogous image denoising method defined by Ghazel *et al.*, we restored the fractal code of the noise-free image from the corresponding noisy image with additive white Gaussian noise (AWGN) for general upscaling. *λ_i_* is the sum of the noise free density value *λ̃_i_* and noise *e_i_*, where *e_i_* is independent, identically distributed (*i.i.d.*) and drawn from a normal distribution with mean 0 and variance $\delta_{\text{e}}^{2}$, and *e_i_* is not correlated with *λ̃_i_*:

(11) $$\lambda_{i}={\tilde{\lambda }}_{i}+e_{i},e_{i}∼N(0,\delta_{e}^{2})$$

From Equations (10) and (11):

(12) $$v=\sum_{i=1}^{n\times n}\omega_{i}({\tilde{\lambda }}_{i}+e_{i})=\tilde{v}+\sum_{i=1}^{n\times n}\omega_{i}e_{i}$$

The symbol “∼” denotes the noise-free counterpart to be estimated. Since *e_i_* is an independent and identically-distributed random variable, and is not correlated with *λ̃_i_*, the relationship of the mathematical expectation and variance between *v* and *ṽ* can be represented in the form:

(13) $$E(v)=E(\tilde{v}+\sum_{i=1}^{n\times n}\omega_{i}e_{i})=E(\tilde{v})$$

(14) $$\delta_{v}^{2}=\text{Var}(\tilde{v}+\sum_{i=1}^{n\times n}\omega_{i}e_{i})={\tilde{\delta }}_{v}^{2}+\sum_{i=1}^{n\times n}\omega_{i}^{2}\cdot \delta_{e}^{2}$$

where *E*(*v*) and *E*(*ṽ*) are mathematical expectations of *v* and *ṽ*, respectively; and $\delta_{\text{v}}^{2}$ and ${\tilde{\delta }}_{\text{v}}^{2}$ are mathematical variances of *v* and *ṽ*, respectively. After estimating the least-squares coefficients *α* and *β* from Ghazel *et al.* (2003), let *σ* = Σ*ω_i_* (*i* = 1, 2, …, *n*^2^), then:

(15) $$\alpha =\text{Cov}(X,Y)/\delta_{X}^{2}=[\text{Cov}(\tilde{X},\tilde{Y})/{\tilde{\delta }}_{X}^{2}]/[1+\sigma \cdot \delta_{e}^{2}/{\tilde{\delta }}_{X}^{2}]$$

where the image is regarded as a random field, and X and Y are random variables representing the density value distribution of the upscaled domain block and the range block, respectively. The numerator is the noise-free version *α̃* of *α*. Thus, the noise-free image fractal code parameters *α̃* and *β̃* are:

(16) $$\tilde{\alpha }=(1+\sigma /\gamma )\alpha ,\tilde{\beta }=E(Y)-\tilde{\alpha }E(X)$$

where $\gamma ={\tilde{\delta }}_{X}^{2}/\delta_{e}^{2}$ is the signal-to-noise ratio. Meanwhile, the corresponding collage error is represented as:

(17) $$\begin{array}{l} {\tilde{\Delta }}_{ij}^{\left(k\right)}=E[{\left(({\tilde{\alpha }}_{ij}{\tilde{X}}_{j}^{\left(k\right)}+{\tilde{\beta }}_{ij})-{\tilde{Y}}_{i}\right)}^{2}]\\ \;={\tilde{\alpha }}_{ij}^{2}(E[{\left(X_{j}^{\left(k\right)}\right)}^{2}]-\sigma \delta_{e}^{2})+2{\tilde{\alpha }}_{ij}{\tilde{\beta }}_{ij}E[X_{j}^{\left(k\right)}]+{\tilde{\beta }}_{ij}^{2}-2{\tilde{\alpha }}_{ij}E[X_{j}^{\left(k\right)}Y]-2{\tilde{\beta }}_{ij}E[Y]+(E[Y_{i}^{2}]-\delta_{e}^{2}) \end{array}$$

Then, the noise-free image can be restored from the noisy image with PIFS coding.

#### 2.2.4. Fractal decoding and downscaling

The set of five elements (*i, j, k, α̃_ij_, β̃*_ij_) constitutes the PIFS code of a noise-free image *Ĩ*. The PIFS code depicts the fractal self-similarity and self-affinity from which a fractal image can be decoded at different scales with different magnification/minification factors. Then, fractal decoding-based downscaling is done by iterating on an arbitrary initial image (e.g., a blank image), with the PIFS code and a magnification factor greater than one, until the destination SR image is stable and unchanged. The convergence is guaranteed by the contractive mapping fixed-point theorem. In practice, the Euclidian distance is measured between adjacent iteration results. For example, a distance less than 1 × 10^-6^ could be acceptable as little difference between two images when the gray level lies in [0,255].

### 2.3. Parameter Estimation

**Figure 4.**
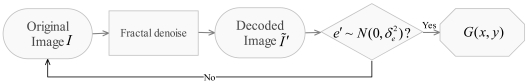
Work process to estimate the ITF parameter.

In the real world, different natural scenes/phenomena have different upscaling information transfer rules and formulisms. Even though some real scenes follow the rule of Gaussian upscaling information transfer model between scales, they may have different Gaussian function distribution intensities. The Gaussian upscaling function's form, then, would affect the reconstruction result directly. In the absence of *a priori* knowledge of the image, we resort to blind estimation for the real Gaussian upscaling form. If *Ĩ* is the noise-free image and *I* is the observed image with AWGN noise *e* with mean 0 and variance $\delta_{\text{e}}^{2}$, then *I* = *Ĩ* + *e*. There are two unknown parameters in the expression, viz.: the variance $\delta_{\text{e}}^{2}$ of AWGN noise and the variance $\delta_{\text{g}}^{2}$ of the Gaussian upscaling function. Estimating the two parameters is a precondition for enhancing the resolution of the image. It is well known that in the real world there are many small regions with uniform pixel values, and thus diminutive variation in these regions are mainly caused by noise [26]. Based on this assumption, the variance $\delta_{\text{e}}^{2}$ can be estimated from the local statistics of block pools that consist of all possible small regions in the image. The variance $\delta_{\text{g}}^{2}$ is estimated with a search method based on the assumption that the error image *e* (*e* = *I* - *Ĩ*) is a random field of white Gaussian noise (WGN) with mean 0 and variance $\delta_{\text{e}}^{2}$ (Figure 4). First, the range of $\delta_{\text{g}}^{2}$ is estimated for the search process. For a selected variance, we reconstructed the corresponding denoised image *Ĩ*′ with the proposed fractal coding method. Then, an error image *e*′ (*e*′ = *I* - *Ĩ*′) is generated and checked to see whether it followed the WGN distribution with mean 0 and variance $\delta_{\text{e}}^{2}$. The most appropriate value could be selected to estimate the Gaussian ITF variance. Especially, in the absence of noise in *I*, the goal of the search process is to minimize the difference between *Ĩ*′ and *I* as expected.

## 3. Empirical Study

**Figure 5.**
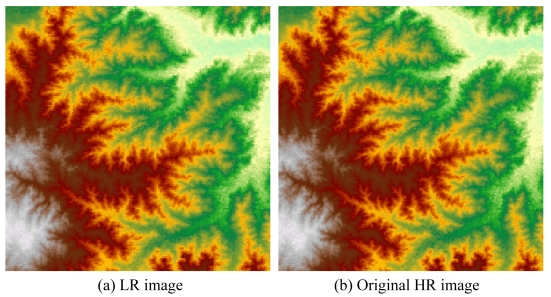
SRTM elevation dataset.

Shuttle Radar Topography Mission (SRTM) is a joint project between NASA, NGA, and the German and Italian Space Agencies to obtain a global digital topographic dataset [32]. In the United States, there are two kinds of SRTM elevation datasets, namely, 1 arc-second (approximately 30 m) and 3 arc-second (approximately 90 m) of latitude and longitude. We reconstructed a SR image from a 3 arc-second SRTM elevation dataset, and compared it with the original high-resolution (HR) (1 arc-second) image (Figure 5). The LR image size is 180 pixels by 180 pixels. The research region is located at 35.48N-35.63N, 99.68W-99.53W.

### 3.1. Multifractal Characteristic

**Figure 6.**
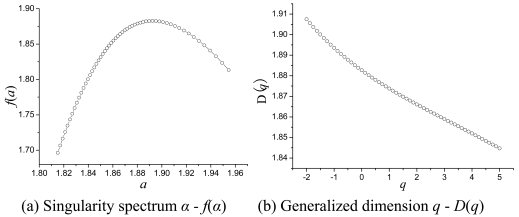
Multifractal spectrum of SRTM.

The singularity spectrum *α - f*(*α*) quantitatively elucidates the relationship of the singularity strength *α* and Hausdorff dimension with the multifractal measure. To calculate the singularity spectrum and generalized dimension, the image was partitioned into boxes of size *l*, where *l* = 2, 4, 8, 16, 32, 64 and 90 pixels. The range of the moment order *q* was from -2 to 5 with steps of 0.125, which was fine enough to show the multifractal characteristic of the image. *R*^2^ of all linear fits were equal to or greater than 0.99. Some characteristics of the spectrum were used to estimate a measure's multifractality (Figure 6a). The multifractal spectrum was obtained by the method developed by Chhabra *et al.* [23]. A typical multifractal spectrum is a single-hump, convex curve reaching its maximum at *α*_0_ (*q* = 0, *q* is the moment order). The *f*(*α*) spectrum at the left and right of the maximum corresponds to *q* > 0 and *q* < 0, respectively. Large values of |*q*| correspond to large distortions of the measure magnifying its large (*q* > 0) or small (*q* < 0) concentrations. The maximum was reached at *q* = 0, and then the magnitude decreased significantly around when *q* > 0 and *q* < 0 (Figure 6b), which showed that the SRTM image was heterogeneous and had a multifractal characteristic. This was validated by the width of the singularity spectrum, which indicates the range of the Lipschitz-Holder exponent *α* in the image. For a mono-fractal image, the *f*(*α*) spectrum surrounds the maximum value *f*(*α*_0_), and the spectrum width is small and tends toward 0. An image with a convex and wide singularity spectrum can be considered multifractal rather than mono-fractal.

The range of the singularity strength *Δα* is a difference of the maximum and minimum *α* when *f*(*α*) > 0, Δ*α* = *α_max_ - α_min_*, which represents the extent of a possible Lipschitz-Holder exponent [33]. *α*_min_ and *α*_max_ are calculated by fitting the spectrum curve and taking the point of intersection with the *α*-axis. *Δα* makes a quantitative measurement of the degree of multifractality. The broader the spectrum *Δα*, the stronger the multifractality, and the richer and more complex the image pixel intensity distribution is. *Δα* of the SRTM image is about 0.53, which indicates the presence of a strong degree of multifractality in the image.

Asymmetry of the *α–f*(*α*) spectrum shape is also an important index of multifractality. It indicates the degree of fluctuation in different fractal exponents [34]. A left-skewed spectrum denotes high fractal exponents and large fluctuations dominate the measure, while a right-skewed spectrum implies low fractal exponents and low fluctuations are dominant. To estimate the skewness quantitatively, the degree of asymmetry was calculated with the formula *A* = (*α*_0_ - *α*_min_)/(*α*_max_ - *α*_0_) [34,35], where *A* = 1 if the spectrum is symmetric and *A* > 1 or *A* < 1 when the spectrum is left-skewed or right-skewed. The spectrum shape of the SRTM image was right-skewed and the degree of asymmetry was about 0.89, which indicates that both large and small fluctuations are present and small fractal exponents dominate rather than high ones.

### 3.2. Super-Resolution Images

**Figure 7.**
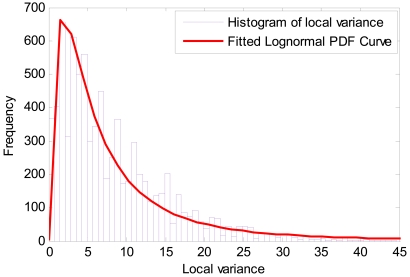
Probability distribution function of local variance.

To estimate the noise variance of the image, a local statistics method was adopted. A moving window with a size of 2 by 2 pixels was selected to collect all possible blocks in the image, which moved left-to-right and top-to-bottom, and the displacement was one pixel at a time. Then, a histogram of the local variance distribution was generated, which approximately followed a lognormal distribution with mean 1.72, variance 1.03 (Figure 7), and the standard error of the estimated mean and variance are about 0.011 and 0.008, respectively. The coefficient of determination (*R*^2^) of curve fit is 0.93 (*p* < 0.01). The most frequent value in the distribution, 1.7, was taken as the noise variance of the SRTM image. The variance $\delta_{\text{g}}^{2}$ was estimated with a search method based on the assumption of AWGN. The sizes of the range block and domain block were 2 by 2 pixels and 6 by 6 pixels, respectively, and the size of the Gaussian template was 3 by 3 pixels. The range estimated for the noise variance $\delta_{\text{g}}^{2}$ was (0.2, 2) since, for a discrete Gaussian template of 3 by 3 pixels, the density distribution of the template whose variance is lower than 0.2 or higher than 2 is almost unchanged. The most appropriate variance estimated was about 0.8.

**Figure 8.**
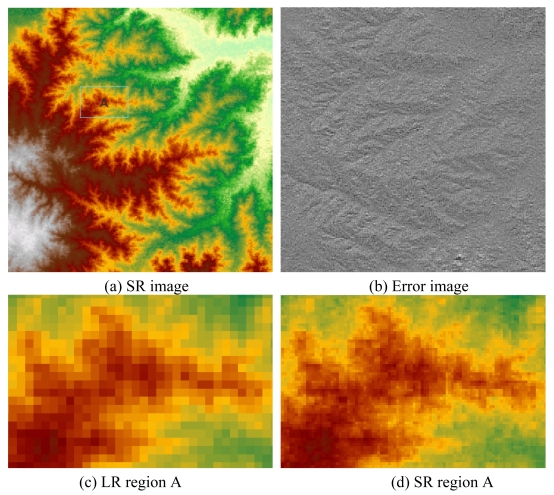
Super-resolution reconstruction of SRTM image.

After estimating the AWGN noise and information transfer function *s*, the PIFS code of the LR SRTM image was generated with the proposed method. Then, we reconstructed a SR version of the image with 3x-enhanced spatial resolutions (1 arc-second by 1arc-second per pixel) (Figure 8a). The original LR image is much coarser than the original high resolution image because of its low spatial resolution. In the SR image, many details were added compared to the LR image (Figures 8c, d). The error image was the difference between the estimated SR image and the original real high-resolution image (Figure 8b). The mean and standard deviation of the error image were 0.09 m and 2.25 m, respectively.

The SR image is an estimation of the real scenes. It performs quite well in recovering details. At the same time, there was also a bit of blur and block in some regions of the SR image (Figure 8d). From the relationship *Ĩ* = *I* − *e*, the blur is tied in with the estimated noise *e*. Accurate estimation of *e* brings the real *Ĩ*, while any bias of the estimated *e* would generate a distorted version of *Ĩ*. In the research, the noise *e* was estimated with a local statistics method from just one image. More *a priori* knowledge and data about the image would be helpful to improve the effect.

## 4. Discussion and Conclusions

Remote sensing provides an important approach to investigating the earth, but the spatial resolution is usually very low when researching at relatively small spatial scales, such as an intra-urban scale, rather than continental or national scales. In this paper, a fractal-based super-resolution reconstruction method was introduced. Self-similarity or self-affinity presented between different scales makes it possible to reconstruct details at a smaller scale than the original LR image's scale. We explored self-similarity and self-affinity characteristics with a multifractal analysis method. Singularity spectrum and generalized dimension are efficient indices to measure the self-similarity and self-affinity characteristic of the image. Multifractality is common in nature, especially geophysics. Different phenomena have different information transfer mechanisms between scales. The ITF determines how information is transferred and lost in upscaling. In the absence of *a priori* knowledge, the ITF is estimated with a Gaussian model, which is a generalization of downsampling and averaging methods. A search strategy was adopted to estimate the ITF parameter. This method worked well, although it is somewhat time consuming. In the presence of AWGN, the relation between a noise-free image and noisy image is connected by a PIFS code, which can be used to reconstruct a noise-free SR image. Most of the process is completed with fractal coding. Then, a fractal-based denoising and spatial resolution enhancement method was developed to reconstruct super-resolution images of the SRTM elevation dataset. Analysis results showed that the proposed method is efficient and performed well in reconstructing super-resolution images for the dataset. Satellite earth observation is a typical example of the application of the general SR reconstruction method. The analysis and operation process of the proposed method is also applicable to other images with multifractal characteristic.

How to estimate the AWGN *e* and IFS *s* efficiently are two important issues. A good search strategy will reduce processing time. More auxiliary data and *a priori* knowledge would be helpful to improve the effect, which will be studied in future research. Furthermore, besides additive white Gaussian noise *e* [in Equation (1)] which was explored in this paper, there may be other kinds of noise, such as nonlinearly added noise. The proposed denoising and SR reconstruction method is not adaptable for this case. How to reconstruct SR images in non-AWGN cases is also an interesting and important problem.
